# Classrooms' indoor environmental conditions affecting the academic achievement of students and teachers in higher education: A systematic literature review

**DOI:** 10.1111/ina.12745

**Published:** 2020-10-21

**Authors:** Henk W. Brink, Marcel G. L. C. Loomans, Mark P. Mobach, Helianthe S. M. Kort

**Affiliations:** ^1^ Research Centre for Built Environment NoorderRuimte Hanze University of Applied Sciences Groningen The Netherlands; ^2^ Department of the Built Environment Building Performance IEQ‐Health Eindhoven University of Technology Eindhoven The Netherlands; ^3^ Research Group Spatial Environment and the User The Hague University of Applied Sciences The Hague The Netherlands; ^4^ Research Group Technology for Healthcare Innovations Research Centre Sustainable and Healthy Living Utrecht University of Applied Sciences Utrecht The Netherlands

**Keywords:** academic performance, facility management, indoor environmental quality, quality of teaching and learning, school design

## Abstract

This study reports the outcomes of a systematic literature review, which aims to determine the influence of four indoor environmental parameters — indoor air, thermal, acoustic, and lighting conditions —on the quality of teaching and learning and on students' academic achievement in schools for higher education, defined as education at a college or university. By applying the Cochrane Collaboration Method, relevant scientific evidence was identified by systematically searching in multiple databases. After the screening process, 21 publications of high relevance and quality were included. The collected evidence showed that the indoor environmental quality (IEQ) can contribute positively to the quality of learning and short‐term academic performance of students. However, the influence of all parameters on the quality of teaching and the long‐term academic performance could not be determined yet. Students perform at their best in different IEQ conditions, and these conditions are task‐dependent, suggesting that classrooms which provide multiple IEQ classroom conditions facilitate different learning tasks optimally. In addition, the presented evidence illuminates how to examine the influence of the IEQ on users. Finally, this information supports decision‐makers in facility management and building systems engineering to improve the IEQ, and by doing so, allow teachers and students to perform optimally.

**Practical Implications**

- Over the last decades, research has shown that classroom conditions in schools are far from optimal. In some cases, conditions can even be unhealthy and affect teachers' and students' performance adversely. When students do not perform to the best of their ability, this can have serious consequences for the individual and society.
- European and Dutch authorities recognize the need for better classrooms and stress its urgency. The gathered evidence in this study helps all involved to understand the extent of the influence of the IEQ on educational processes and outcomes. It can form the basis for better‐informed decision‐making, especially for those who are involved in renovation or new construction of school buildings for higher education.
- Specifically, facility management (FM) and building‐related engineering partners of FM can use this information to design a more user‐oriented built environment. By doing so, this increases the likelihood of classrooms in higher education supporting educational outcomes. Moreover, this potentially allows future generations of teachers and students to perform and learn better in a healthier environment.

## 1 INTRODUCTION

In schools, students learn to form positive social relationships, gain independence, and develop emotionally, behaviorally, and cognitively.^1^ The most important role for school management is to provide an optimal school climate that represents virtually every aspect of the school experience. This includes the quality of teaching and learning, school‐community relationships, school organization, and the institutional and structural features of the school environment.^2^ This is also a challenge in the education at a college or university, hereafter referred to as higher education.^3^ To facilitate the school climate, higher education school management provides buildings, assets, and services for their employees and students. The role of facility management (FM) is to coordinate and maintain these assets and services.^4^ By doing so, FM influences a school's ability to act proactively and meet all requirements to create a positive school climate.^4^ A positive school climate is associated with students' healthy development and the retention of teachers, and can even have a predictive value for the academic achievement of students.^1^ In order to improve the effectiveness of this climate, FM has an active role in creating an optimal learning environment. This requires, among other things, appropriate ventilation, heating and air conditioning, ample forms of lighting, necessary acoustical control, and upkeep of maintenance.^2^

**FIGURE 1:**
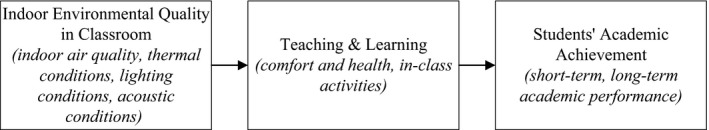
Conceptual framework for the influence of indoor environmental conditions on academic achievement in schools^2^, ^7^, ^8^

This study focusses on the indoor environment, which is a system of the indoor air quality (IAQ), thermal conditions, acoustic conditions, and lighting conditions.^5^ Many factors may influence the academic achievement of students,^6^ but the indoor environmental quality (IEQ) in classrooms can potentially influence teaching and learning positively,^7^, ^8^ which in turn increases the likelihood of a better academic achievement of students (Figure 1).^2^

The IEQ addresses the subtle issues that influence how users experience an indoor space, for example, a classroom.^9^ The IEQ results from a variety of pollutants or other determinants that can be caused by all four indoor environmental parameters. In this context, occupants' comfort depends on the actual indoor environmental conditions and personal demographic characteristics, such as gender and age.^5^ In addition, it depends on psychobiological processes, such as arousal and stress, and psychological processes, such as perceived control and attention.^10^ Moreover, the IEQ to which teachers and students are exposed, can affect teaching effectiveness and instructional practices,^7^ which in turn can affect students' academic achievement.^2^ A study of Kok et al^11^ showed, for example, a statistically significant positive relationship between teachers' perceptions of classrooms' lighting and acoustic conditions and study success. Also, the IEQ can influence users' task performance, communication and social interaction, mood, and health and safety at school.^2^, ^5^, ^12^ This influence has often been examined by analyzing the effect of one parameter.^5^ In 2016, Wargocki and Wyon^8^ analyzed the combined effect of thermal comfort and IAQ on humans' short‐term cognitive performance. These researchers identified the following human mechanisms which are affected by both thermal and IAQ conditions: distraction and attention, motivation, arousal, neurobehavioral symptoms, and acute health symptoms. Moreover, lighting conditions, for example, can affect mental alertness and cognitive performance^13^; and annoyance and distraction can be caused by poor acoustic conditions.^14^ Furthermore, a poor IEQ can cause adverse health outcomes, which can cause sick leave and impaired academic achievement.^15^ Students' academic achievement also depends on how teachers use all resources to improve in‐class activities.^2^ Finally, it depends on the students' ability to concentrate and think clearly, as these aspects together influence the in‐class academic performance of students.^8^ Therefore, to assess both the individual and the combined influences of all four indoor environmental parameters on the quality of teaching and learning and students' academic achievement is a worthwhile exercise.

At this moment, there are no specific guidelines available for higher education school buildings. The focus of earlier research, for example addressing the IAQ, was mainly on pupils in primary and secondary education.^16^ Based on the outcome of this research, specific IEQ guidelines for pupils of primary and secondary schools were developed.^17^ However, facilitating young adults (aged 18‐25 years) and teachers (aged 25‐65 years) in higher education might require a different IEQ in which they can perform optimally. In order to support initiatives, which aim to develop specific IEQ guidelines for higher education school buildings, this review aims to provide an overview of how classroom indoor environmental conditions influence the quality of teaching and learning and students' academic achievement in higher education.

The following research question is explored in this review: What is the effect of IEQ in classrooms in higher education on the quality of teaching and learning, and students' academic achievement? In addition, three hypotheses will be examined: (a) the IEQ influences the quality of teaching; (b) the IEQ influences the quality of learning, and (c) the IEQ influences the students' academic achievement. In addition, in the context of this study, the quality of teaching and learning is operationalized by how teachers and students perceive teaching quality, learning quality, and their physical and mental health. Students' academic achievement refers to their short‐term and long‐term academic performance.^2^ Short‐term academic performance is often quantified with cognitive performance tests or with the use of school exercises.^8^, ^18^ Long‐term academic performance focusses on the performance of students for a course or academic year.^19^, ^20^

## 2 METHODOLOGY

We applied the Cochrane Collaboration Method to identify relevant literature for review.^21^ We included laboratory experiments and field experiments; results obtained in both settings can reveal how the IEQ influences the quality of teaching and learning and students' academic achievement. Included were studies that addressed students and teachers in higher education with no physical disabilities (eg, diseases, blindness, and under sedation) or mental disabilities (eg, auditory processing disorder, attention disorders, and dyslexia) and that are written in English. In addition, we included studies that addressed the physical environmental conditions in combination with physiological conditions (eg, attention, comfort, discomfort, illness, stress, and vitality), affective responses (eg, perceived mood and emotions), or the influence on teaching, learning, or academic achievement. We did not apply any restrictions with regard to the publication year and searched relevant databases until the 20th of May 2020. We identified potentially relevant literature through computerized searches in the following databases: Web of Science, Scopus, Emerald Insight, Wiley Online Library, Sage, PubMed, and 27 EBSCOhost databases (ie, Academic Search Premier, ERIC, APA PsycINFO, Teacher Reference Center), which were searched simultaneously. For the search, we used keywords that are related to classrooms in higher education, IEQ, teaching, learning, and students' academic achievement. Appendix 1 presents an overview of the used keywords during the search. The used search strings can be found in Appendix 2.

The search through the selected databases yielded 2501 publications, which were imported in RefWorks. After removing duplicates (n = 608), we analyzed the relevance of the selected publications. When the title, keywords, or abstract did not give any indication that indoor environmental conditions were studied, the publication was excluded (n = 1512). These publications emerged in the primary search because one or more keywords were used in a different context. For instance, a study used the word “light” or “noisy” as an adjective, or the word “illuminate” was used as a synonym for “illustrate” or “embellish.” We also excluded publications that addressed only the physical indoor environmental conditions, or other types of building performance (eg, energy consumption and sustainability) without analyzing the effect on teaching, learning, or academic achievement (n = 135). Finally, we excluded publications that addressed humans with physical or mental disabilities (n = 44), and publications that did not address classrooms in higher education (n = 131). All publications not written in English were excluded (n = 8). In total, 63 publications were included after this selection stage.

As a final selection stage, the relevance and quality of the 63 remaining publications were determined. To assess the relevance of the included publications, the context and scope of the study were assessed. The context of the study was high when the influence of multiple indoor environmental conditions on the quality of teaching, learning, or academic achievement was analyzed. In addition, the scope of the study was high if the study analyzed indoor environmental conditions and assessed the impact of these conditions on the performance of teachers or students. Moreover, the reliability and the methodological quality of the study were analyzed to assess the quality of the study. The reliability of the study was high when it was published in a peer‐reviewed journal and provided detailed information about the sample (eg, sample size, gender, age, and standard deviation). The methodological quality was high when the methodological section in the study described in detail how the research was conducted and when the applied tests or questionnaires were available (eg, appendix and website). In addition, this quality was high when the study provided detailed information about the accuracy of used measuring equipment and how the measurements were performed in the classroom (eg, position, number of measurements, and measuring height). Finally, the methodological relevance was high when three or more key performance indicators of the targeted four indoor environmental parameters were measured, because these studies may reveal, in particular, the combined influence of the indoor environment.

**FIGURE 2:**
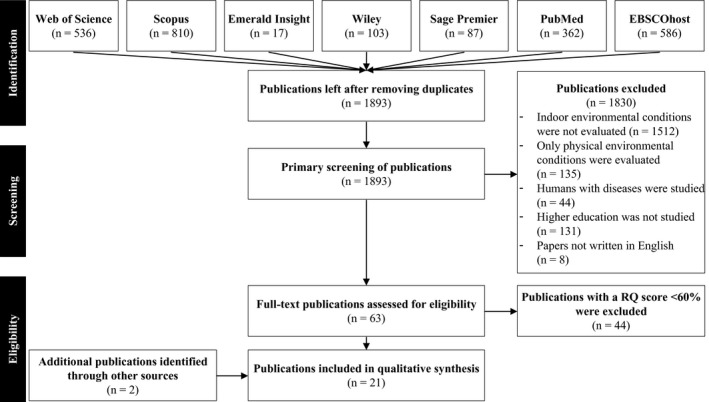
Flowchart of the screening process of the literature

Independently, the authors scored publications, compared the individual scores, and adjusted the rubrics of the assessment tool. Appendix 3 presents the authors' final version of the assessment procedure, which was used for scoring the relevance and quality of all publications. The context, scope, reliability, and methodological quality scores were expressed in a percentage of the maximum score (100 percent). Studies with a relevance and quality (RQ) score lower than 60 percent were excluded (n = 44). Through hand‐searching, using the title of the study in Google Scholar, two additional studies have been identified. These studies addressed the same research as the, through the systematic search, identified publication; however, they contain additional and relevant information. The first study added is the doctoral thesis of Ahmed^22^ and complements the study of Ahmed et al^23^ The second study is of Mishra et al^24^ and complements the study of Kooi et al^25^ Figure 2 summarizes all different selection stages during the screening process, which eventually led to the identification of the 21 included studies.

If an included study examined students' and teachers' comfort and health, this is linked to the quality of teaching or learning. Examined students' cognitive performance, for example, attention or concentration tests, and students' score on school tests, for example, calculation and reading tests, was classified as short‐term academic performance. Students' grades of a course or academic year were classified as long‐term academic performance. Reported statistical significant effects of different IEQ conditions on students comfort, health, and academic achievement were included in the analyses. In addition, reported statistical significant effects on students' academic achievement were quantified by calculating the increase or loss of the reported performance, based on the scores presented.

## 3 RESULTS

**FIGURE 3:**
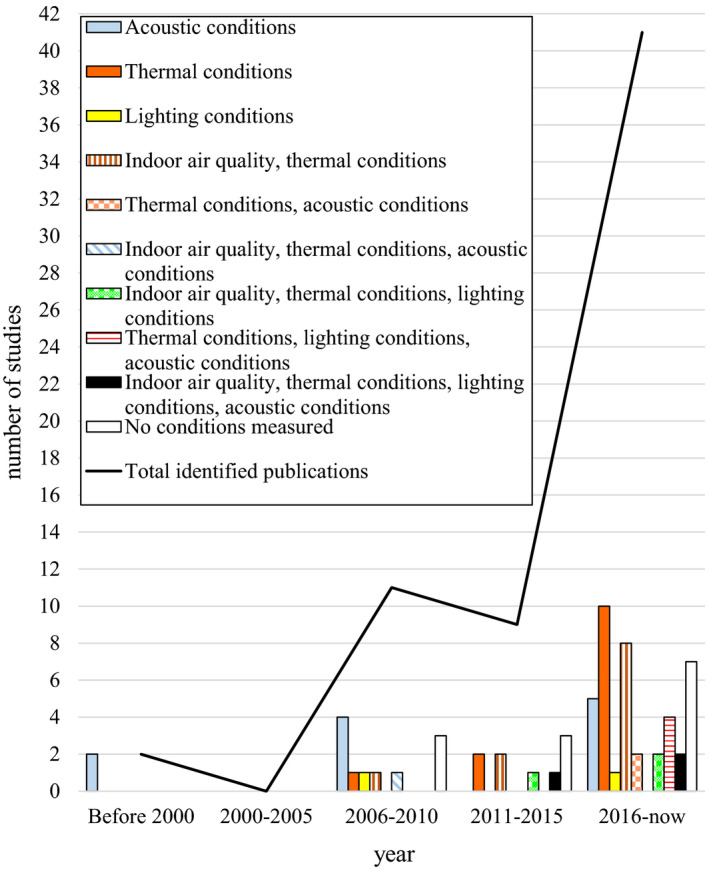
The number of studies addressing the IEQ in classrooms in higher education, the distribution over the indoor environmental parameters, and the distribution over the years

Figure 3 shows the development in time of the distribution of the 63 identified studies, before the final selection stage and the distribution with respect to the studied indoor environmental parameters. The number of identified publications in relation to the year of publication indicates a growing interest in the influence of the IEQ on learning and academic achievement. Three studies addressed all four environmental parameters on the quality of teaching, learning, or academic achievement. The results of this review were derived from 19 identified and two additional studies of high quality and relevance with a RQ‐score of 60% of higher.

Tables 1 and 2 describe key features of the 21 included studies. We derived the table layout of table 1 from the way Mendell and Heath^15^ presented their results in 2007. Table 1 presents the direct associations between indoor environmental conditions and students' academic performance. Table 2 presents direct associations between actual or perceived indoor environmental conditions and perceived academic performance, physical health, or comfort.

**TABLE 1:** Findings from research on direct associations between indoor environmental conditions and students' academic performance. See footnote to table for the explanation of all variables and symbols used

Outcome	Study features							Effect of indoor environmental parameter								Reference
								TC		IAQ		AC		LC		
	Geographic location	Setting	Subject	N	Design	Key confounders	RQ‐score in %	Higher temperature	Lower temperature	Higher quality	Lower quality	Higher quality	Lower quality	Higher quality	Lower quality	Author
Accuracy in complex, and vigilance tasks	Saudi Arabia, (Jeddah)	cc	♀ S	499	C	@	92	**↓**			**↓**					Ahmed^22^, ^23^
Accuracy in memory tasks								**↓**	**↑**		**↓**					
Concentrated and distributive attention test	Romania (Timisoara)	cc	♀♂ S	18	qE	@	85	**↓**	**↑**		**↓**					Sarbu^26^
Distributive attention test								**↓**	**↑**		**↓**					
Number of hits in performance test	Brazil	cc	♀♂ S	84	qE	@	85	**↕**								Siqueira^27^
Time spent on performance test								**↑**								
Recognition rate	China	l	♀♂ S	8	qE	@	85							**↓**		Yan^29^
Perception test	China	cc	♀♂ S	10	qE	@	85	**↓**	**↓**				**↓**	**↑**		Xiong^28^
Memory test								**↑**					**↓**			
Problem‐solving test								**↓**	**↓**				**↓**	**↑**		
Attention‐oriented test									**↑**				**↓**	**↑**		
Short‐term memory and verbal ability test	Italy (Lombardia)	cc	♀♂ S	20	qE	@	81	**↓**								Barbic^31^
Reasoning test								**↕**								
Knowledge test (examination score)	USA (Amherst)	c	♀♂ S	409	C	@	79	**↓**	**↓**							Hoque^33^
Perception test	Saudi Arabia (Riyadh)	l	S	40	qE	@	79	**↓**	**↓**							Almaqra^34^
Lexical decision test	USA	cc	♀♂ S	158	E		69						**↓**			Shelton^37^
Knowledge test													**↓**			
Knowledge test	USA	cc	♀♂ S	71	E		66						**↓**			End^38^
Attention test	Colombia (Bogotá)	cc	♀♂ S	141	qE		60						**↓**			Castro‐Martínez^42^

Note

Geographic location: country (place or region if reported)

Setting: c = classroom, cc = environmental condition controlled classroom, l = laboratory or climate chamber.

Subject: ♀ = female participants, ♂ = male participants, S = students, T = teachers.

Design: E = experiment, qE = quasi‐experiment, C = cohort.

RQ: relevance and quality score in %.

Key confounders: @ = key confounders are controlled

Effect of indoor environmental parameter: **↓ **= negative effect, **↑ **= positive effect, **↕ **= no effect, red marking: negative effect (*P* ≤ 0.05), green marking: positive effect (*P* ≤ 0.05), no marking: no statistical significant effect (*P* > 0.05 or not reported).

Correlation: += positive correlation ‐= negative correlation green signifies included and measured ^*^ = *P* ≤ 0.05, ^**^ = *P* ≤ 0.01, ^***^ = *P* ≤ 0.001,^○^= *P* > 0.05, ^x^ = not reported or no correlation.

**TABLE 2:** Findings from research on direct associations between actual or perceived indoor environmental conditions and perceived academic performance, physical health, or comfort

Outcomes	Study Features							Association		Reference
	Geographic location	Setting	Subject	N	Design	Key confounders	RQ‐score in %	Effect	Correlation	Author
Accuracy in cognitive performance tasks	Saudi Arabia, (Jeddah)	cc	♀ S	499	C	@	92	Thermal sensation slightly warm, cool, and slightly cool have a positive effect on the outcome compared to thermal neutral sensation	+^***^	Ahmed^22^, ^23^
								Thermal sensation cold and thermal discomfort that attributes to inability to focus and numbness in fingers have a negative effect on the outcome compared with thermal neutral sensation	‐^***^	
								Reported symptoms of headache, dizziness, heaviness on head, confusion, difficulty thinking, difficulty concentrating, and fatigue have a negative effect on the outcome compared with no reported symptoms	‐^***^	
Heart rate	Brazil	cc	♀♂ S	84	qE	@	85	An air temperature of 27.95°C (globe temperature 25.50°C) increases the heart rate compared with an air temperature of 22.60°C (globe temperature 23.11°C)	+^*^	Siqueira^27^
Error rate and cognitive performance								Thermal discomfort has a negative effect on the outcome compared with a thermal neutral sensation	‐^*^	
Blood pressure								Thermal discomfort has a negative effect on the outcome compared with a thermal neutral sensation	‐^*^	
Saliva cortisol concentration	Sweden (Helsingborg)	cc	♀♂ S	72	qE	@	83	The effect of a LED lighting system compared with a T5 lighting system on saliva cortisol concentration	+^*^^/ x^	Gentile^30^
Perceived mood and light perception								The effect of a LED lighting system compared with a T5 lighting system on perceived strength and quality of lighting conditions	+^***^	
Heart rate	Italy (Lombardia)	cc	♀♂ S	20	qE	@	81	Thermal discomfort has a negative effect on the outcome compared to a thermal neutral sensation	‐^***^	Barbic^31^
Melatonin concentration in blood and subjective perception of sleepiness	Republic of Korea (Daejeon)	cc	♀♂ S	15	qE	@	79	The effect of exposure to blue‐enriched white light with a color temperature of 6,500K at an illuminance level of 500 lux causes a decline of the outcome compared with exposure to warm white light with a color temperature of 3,500 K at an illuminance level of 500 lux	‐^*^	Choi^32^
Perceived alertness, mood, and visual comfort								The effect of exposure to blue‐enriched white light with a color temperature of 6,500K at an illuminance level of 500 lux has a positive effect on the outcome compared to exposure to warm white light with a color temperature of 3,500 K at an illuminance level of 500 lux	+^*^	
Cortisol concentration in blood								No effect was observed on the outcome if participants were exposed to blue‐enriched white light with a color temperature of 6,500K at an illuminance level of 500 lux compared to when they were exposed to warm white light with a color temperature of 3,500 K at an illuminance level of 500 lux	^‐^	
Examination scores	USA (Amherst)	cc	♀♂ S	409	C		79	Perceived thermal comfort has a positive effect on the outcome compared to perceived thermal discomfort	+^*^	Hoque^33^
Perceived concentration and productivity loss	Serbia (Belgrade)	cc	♀♂ S	240	qE	@	76	Perceived local thermal discomfort caused by radiant asymmetry or vertical air temperature difference has a negative effect on the outcome compared with perceived thermal neutral sensation	‐^x^	Bajc^35^
Actual thermal sensation	The Netherlands (Eindhoven)	c	♀♂ S	384	C	@	71	Even when thermal conditions during class did not change much, thermal perception was different at class entry compared to thermal perception after the first 45 minutes	^*^	Mishra^24^, ^25^
Perceived IAQ	Sweden (Lund)	cc	♀♂ S	232	qE	@	70	A carbon dioxide demand‐controlled variable air flow ventilation system has a positive effect on the outcome compared to a ventilation system with a constant air flow	+^*^	Norbäck^36^
Self‐reported headache and tiredness									+^**^	
Satisfaction with IAQ	China (Xi'an)	c	♀♂ S	992	C		66	There was no relation observed between actual CO_2_ concentration and perceived IAQ	^x^	Liu^39^
Actual thermal sensation								A relationship was observed between actual thermal sensation and perceived IAQ	+^*^	
Fundamental frequency, vocal intensity, percentage of phonation, cycle dose	Brazil (Belo Horizonte)	cc	♀ T	27	qE		65	Noisy conditions of 76.0 dB have a negative influence on the outcomes compared to more quiet conditions at 43.92 dB	‐^***^	Rabelo^40^
Self‐reported academic performance	China (Hong Kong)	cc	♀♂ S	312	C		62	Increasing numbers of complains about the IEQ have a negative effect on the self‐reported academic performance	‐^*^	Lee^41^

Note

See footnote to Table 1 for the explanation of all variables and symbols used.

The results are presented on the basis of the RQ‐score, beginning with the study with the highest score, and include the main findings of the influence of the IEQ on teachers' and students' health and comfort and students' academic performance. Appendix 4 provides additional details on these studies, including information about the age of participants, measured indoor environmental performance indicators, and studied outcomes.

Ahmed et al^22^, ^23^ studied the individual and combined effect of different air temperatures, carbon dioxide concentrations (CO_2_), and perceived thermal sensation on the cognitive performance of 499 female students in Saudi Arabia. Participants were exposed to different indoor environmental conditions in two identical university classrooms. This exposure revealed that air temperature affects the accuracy of tasks differently according to the type of task while cognitive performance in all tasks improved significantly (*P* < 0.001) when CO_2_ levels decreased from 1800 to 600 ppm and from 1000 to 600 ppm. Although students' accuracy in complex and vigilance tasks was the highest at an air temperature of 20°, the highest accuracy for memory tasks was observed at an air temperature of 23°C, compared to 20 and 25°C (*P* < 0.001).

Sarbu and Pacurar^26^ analyzed students' concentrated and distributive attention test scores in relation to air temperature, relative humidity, and CO_2_ concentration. Students' cognitive performance peaked at temperatures between 24 and 26°C, a relative humidity of approximately 60%, and a CO_2_ level of approximately 500 ppm. Although the sample size of this experiment was relatively small (n = 18), the researchers report correlations between the observed indoor environmental parameters and students' cognitive performance.

Siqueira et al^27^ tested the cognitive performance of 84 students by means of five different tests in a computer classroom. In addition, the impact on students' health was analyzed by measuring their heart rate. An average score was calculated for the number of hits and the time required. The results showed that the number of hits was similarly distributed over the 3 days of testing and an average air temperature of 22.60°C (Day 1), 23.24°C (Day 2), and 27.95°C (Day 3). The total time spent decreased significantly (*P* < 0.05) over the experimental period. The researchers reported that this effect could have been caused by the students wanting to leave the uncomfortable warm environment more quickly. The thermal conditions of the environment are factors that may affect cardiovascular parameters. The heart rate of the students increased with 8% (*P* < 0.05) at the end of the cognitive activity at Day 3 compared to Day 1 and with 7% (*P* < 0.05) compared to Day 2. An air temperature of 23.3°C was associated with thermal neutral sensation.

Xiong et al^28^ explored the impact of three indoor environmental parameters, that is, thermal, acoustic, and visual conditions, on learning efficiency in an environment‐controlled university classroom. Five female and five male students were exposed to three different air temperatures, three different desk illuminance levels, and three different noise levels. Students' cognitive performance was measured for each condition with four different cognitive function tests. For the perception‐oriented task, students scored highest at a temperature of 22°C, an illuminance level of 2200 lux, and a background noise level of 50 dBA. The scores of a memory‐oriented task were the highest at 27°C, 300 lux, and 50 dBA. At 22°C, 300 lux, and 40 dBA students scored the highest for a problem‐solving task. The final task, the attention‐solving task, was performed the best at 17°C, 2200 lux, and 40 dBA. The memory‐oriented task was the only experiment in which students' cognitive performance was affected by all three indoor environmental parameters (*P* < 0.05). Analyses of the results showed that cognitive performance can decline with as much as 52%, when conditions were the worst. Table 3 presents an overview of the quantified combined effect, of an intervention IEQ condition compared with the optimal IEQ condition, on cognitive performance tasks.

**TABLE 3:** Overview of combined effects of two or more indoor environmental parameters on cognitive performance. See footnote to Table for the explanation of all variables and symbols used

Task	Optimal IEQ condition				Intervention IEQ condition				Effect (%)	Reference
	IAQ (ppm CO_2_)	TC (°C)	LC (lux)	AC (dBA)	IAQ (ppm CO_2_)	TC (°C)	LC (lux)	AC (dBA)		Author
Average score cognitive performance tasks	600	20			1000	23			12	Ahmed^22^, ^23^
Average score cognitive performance tasks	600	20			1800	25			23	
Memory‐oriented task		27	300	50		22	60	70	47	Xiong^28^
Perception‐oriented task		22	2200	50		27	2200	40	32	
Problem‐solving‐oriented task		22	300	40		22	60	70	42	

Note

Condition: IAQ = Indoor Air Quality, TC = thermal conditions, LC = lighting conditions, AC = acoustic conditions.

Effect: red marking: negative effect (*P* < 0.05).

Yan et al^29^ used students' recognition rate, as an indicator for cognitive performance, to determine possible differences between different fluorescent lighting (color temperatures of 2700, 4000, and 6500K) with the same color rendering index (≥80) and luminous values, which were kept constant between 4050 lumen and 4450 lumen. Although the experiment was not conducted in a classroom and with a relative small sample size (n = 8), the results indicated that of the three color temperature lamps used, the fluorescent lamp of 4000K was the most suitable classroom light source. The best color temperature combination was 4000K for classroom light, matched to 2700K for chalkboard light and compared with the worst combination the average recognition rate was 23% higher.

Gentile et al^30^ compared a direct/indirect T5 lighting system to a new completely indirect LED lighting system, which was installed in four identical school classrooms. Besides the electricity consumption, saliva cortisol concentration, as an indicator of students' health, and mood and light perception, as indicators for students' comfort, of 83 students were analyzed. The perceived strength of lighting of the experimental LED lighting conditions was significantly higher (*P* < 0.001) than that of the control lighting conditions, although the maintained horizontal illuminance level was the same in both lighting conditions. Furthermore, no general effects on the level of the stress hormones, that is, cortisol, were observed over the whole observation period. However, during the dark months, the experimental LED system better‐supported students' stress hormones suppression (*P* < 0.05), but it was not clear whether this effect was caused by the different light source, the light distribution, or a combination of both.

Barbic et al^31^ analyzed the impact of thermal conditions on students' health, comfort, and cognitive performance. Twenty students underwent a continuous single‐lead electrocardiogram recording during a two‐hour lecture, on two different days with different classroom temperatures, respectively, 22.4 and 26.2°C. On the second day, most students experienced thermal discomfort, the difference between Day 1 and Day 2 was significant (*P* < 0.0001). This difference in thermal discomfort on Day 2 led to a decline of cognitive functions short‐term memory (−12%, *P* = 0.007) and verbal ability (−24%, *P* < 0.001). There was no decline of the cognitive function reasoning, on the contrary, there was an improvement of 1%, but this effect was not significant (*P* = 0.92). The researchers did not report any health risks, caused by the experienced thermal discomfort on Day 2. However, this discomfort on Day 2 was associated with a higher cardiac sympathetic modulation, as indicated by higher values of heart rate (+10%, *P* < 0.001), which may have adversely influenced the cognitive performance of the students.

Choi et al^32^ analyzed the impact of different lighting conditions on students' health, by analyzing students' melatonin concentration in blood and their perceived health, by analyzing their mood and sleepiness. In addition, students' perceived visual comfort and cognitive performance, by analyzing their perceived alertness, were collected of 15 students, who participated in this research. The researchers found that blue‐enriched LED light exposure might be an effective potential countermeasure for morning drowsiness and dozing off in class, particularly in schools with insufficient daylight. The researchers reported correlations between blue‐enriched LED light exposure and melatonin concentration in blood, subjective perception of sleepiness, perceived alertness, mood, and visual comfort (*P* < 0.05). From an educational standpoint, however, warm white light has been reported to provide a relaxing environment and support communication. Therefore, the application of blue‐enriched white light requires careful consideration and the authors' advice is to incorporate this light appropriately according to learning activities or to apply an auto‐dimming feature in which the warm white light gradually changes to blue‐enriched white light after its prolonged use during the morning.

Hoque and Weil^33^ examined the thermal environment, thermal comfort, and test scores of 409 students. The aim of this study was to quantify the relationship between the air temperature, humidity, air speed, and perceived comfort and students' test score, as an indicator of their short‐term academic performance. The researchers found that students who felt thermal discomfort performed worse on tests than those with no thermal discomfort (*P* < 0.001). Table 4 presents a summary of the effect of thermal sensation on different tasks and academic test scores.

**TABLE 4:** Effect of thermal sensation on different tasks and academic test scores. See footnote to the table for the explanation of symbols used

Task	Thermal sensation							Reference
	Cold	Cool	Slightly cool	Neutral	Slightly warm	Warm	Hot	Author
Accuracy on vigilance tasks	10%	1.5%	2%	–^a^	7%	12%	16%	Ahmed^22^, ^23^
Memory‐oriented tasks/learning tasks	11%	1%	2%	–^a^	0.5%	14%	22%	
Academic test scores	16%	9%		–^a^	9%		16%	Hoque^33^

Note

^a^ Reference score; red marking: negative effect (*P* < 0.001) green marking: positive effect (*P* < 0.001).

Almaqra et al^34^ analyzed thermal conditions and caffeine intake on students' cognitive performance, by analyzing students' score on a Stroop test, which is a perception‐orientated test. The researchers concluded that an increase of caffeine intake did not significantly improve the cognitive performance of the 40 students, who participated in this study. However, the relationship between air temperature and performance appeared to be nonlinear. Students' cognitive performance peaked at 23°C and declined with 48% at a higher temperature of 30°C, and with 29% at a lower temperature of 16°C (*P* < 0.001).

Bajc et al^35^ measured the short‐term academic performance of 240 students and related this performance to their perceived thermal comfort. To determine possible performance loss, students had to perform a listening exercise. The results indicated that personal feelings regarding thermal comfort in buildings are strongly subjective. In addition, the results indicated that performance and performance loss are not just a function of the predicted mean vote (PMV) index, and no simple relation in real conditions can link productivity loss of students with the PMV index alone.

Mishra et al^24^, ^25^ studied the effect of temporal transitions on the perceived thermal comfort of 384 students. They observed that students' thermal perceptions changed significantly (*P* < 0.05) as the class progressed. In addition, they observed gender differences in thermal sensation. After the transition period of about 20 minutes, the correlation between operative temperature and thermal sensation receded and individual thermal preferences evened out.

Norbäck et al^36^ examined the effect of two different ventilation systems on the perceived comfort and physical health of 232 students. Statistically significant differences, in favor of the variable flow conditions, were observed for immediate perception of air quality (*P* = 0.02), headache (*P* = 0.003), and tiredness (*P* = 0.007) and concluded that the critical level of CO_2_ in classrooms is 800 ppm and the critical operational indoor temperature is 22°C.

Shelton et al^37^ and End et al^38^ investigated the effect of a disturbing noise from within a classroom on students' short‐term academic performance with a knowledge test, covering topics presented in the lecture. The results revealed a significant (*P* < 0.05) decline of as much as 37% of students' performance.^37^

Liu et al^39^ examined students' comfort in naturally ventilated university classrooms, in the north‐west of China. A total of 992 responses were collected during days when the mean outdoor air temperature was about 10°C. The results showed that the thermal neutral temperature was 20.6°C and revealed that only thermal sensation has a significant correlation (*P* < 0.05) with air quality perception.

Rabelo et al^40^ analyzed the impact of noisy classroom conditions on the voice of 27 teachers. Observed was that an increase in background noise of 32 dB causes an increase of the fundamental frequency of teachers' voice of 12% (*P* < 0.001), an increase of vocal intensity of 8% (*P* = <0.001), an increase of the percentage of phonation of 16% (*P* < 0.001), and an increase of the number of vibration cycles of 31% (*P* < 0.001). These results indicate that an increase of background noise increases vocal health risks of teachers.

Lee et al^41^ investigated the relationship between the actual IEQ in university teaching rooms, the perceived indoor environmental comfort, and the perceived short‐term academic performance of 321 students. Correlations were found between the self‐reported academic performance and the number of IEQ complaints of students (*P* < 0.05). Compared to the contribution of the thermal and lighting conditions and IAQ, which contribute similar to the overall perceived IEQ, the acoustic conditions were found to be a relatively sensitive contributor to the overall indoor environmental satisfaction with an almost twice as high coefficient value (*P* < 0.0001).

**FIGURE 4:**
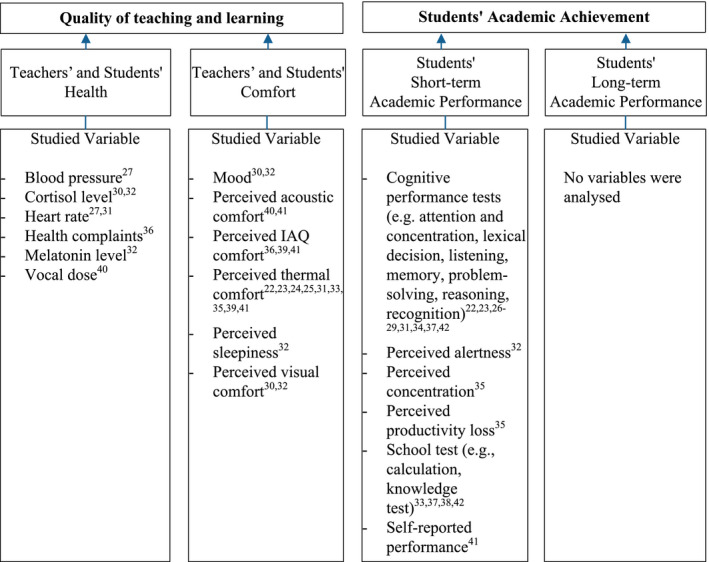
Relation between the studied variables and the quality of teaching, learning, and academic performance

Castro‐Martínez et al^42^ indicated that noise levels have an important effect on the students' attention processes, and that specific changes, aimed at decreasing reverberation values in classrooms (with at least 0.7‐0.9 seconds) affect positively the levels of attention and students' short‐term academic performance. They found that the 141 students, who participated in this study, scored significantly better (*P* < 0.01) on mathematics (+59%), statistics (+18%), and attention (+14%) in a classroom with an average reverberation time of 1.2 seconds compared to students in a classroom with an average reverberation time of 2.0 seconds. Figure 4 presents the relation between the studied variables, presented in the included studies, and the quality of teaching, learning, and students' academic performance.

## 4 DISCUSSION

This review aims to give an overview of 21 studies of high quality and relevance on the influence of the IEQ—as a system of the IAQ, thermal conditions, acoustic conditions, and lighting conditions—on the quality of teaching and learning and students' academic achievement in higher education. Figure 3 shows that in the last decade the IEQ conditions have been examined more holistically and have been conducted in both controlled and free‐running conditions. Only three studies addressed all four indoor environmental parameters.^22^, ^23^, ^41^, ^43^ However, none of these studies analyzed the combined influence of these parameters on the quality of teaching, learning, or academic achievement. Therefore, the emergent properties of all four indoor environmental parameters cannot be determined yet. However, the evidence does illuminate the influence of one or multiple indoor environmental parameters on the quality of teaching, learning, and students' short‐term academic performance. First, we reflect on the influence of the IEQ on the quality of teaching. Secondly, we will discuss the influence of the IEQ on learning. Finally, we will discuss the influence of the IEQ on students' academic achievement.

### 4.1 The influence of the IEQ on the quality of teaching

As explained in the introduction, the quality of teaching is determined by the level of comfort, mental health, and physical health of teachers. Mendell and Heath^15^ relate a poor IEQ to adverse health outcomes and discomfort, which can impair teaching effectiveness and instructional practices,^7^ which in return affect students' academic achievement.^2^ Two publications were identified which addressed the quality of teaching. Therefore, the evidence for the influence of the IEQ on teaching is limited and focusses on one parameter, that is, acoustic conditions. High noise levels in classrooms can cause heavy strain on female teachers' vocal cords and increase teachers' health risks.^40^ However, the intelligibility of a teacher's voice is an essential element in the transfer of knowledge from teacher to student. This is supported by the findings of Castro‐Martínez et al^42^ that acoustics in classrooms could affect the ability of students to hear the teacher. In addition, this ability to hear decreases substantially when the distance between teacher and student increases.^44^ According to Jonsdottir,^45^ voice amplification can improve the intelligibility of a teacher's voice. This researcher reported evidence that voice amplification can positively influence the perceived quality of teaching by teachers, reduce teachers' experienced voice fatigue, and improve student attention. Although this evidence indicates that the acoustic conditions influence the quality of teaching, the amount of evidence is limited to one IEQ parameter, and based on this evidence, the exact influence of all IEQ parameters on the quality of teaching cannot be determined or quantified.

### 4.2 The influence of the IEQ on the quality of learning

The quality of learning, like the quality of teaching, is determined by the level of comfort, mental health, and physical health of students. Mendell and Heath^15^ relate a poor IEQ to adverse health outcomes, discomfort, and distraction of students; the latter negatively influencing students' achievement.

The actual and perceived IAQ can be positively influenced by applying a CO_2_ demand‐controlled ventilation system.^36^ Sufficient ventilation will contribute to maintaining good air quality during the use of classrooms^36^ and will positively influence the perceived overall IEQ.^41^ One study could not find a relationship between the actual CO_2_ concentration and perceived IAQ.^39^ However, a significant correlation was observed between the actual thermal sensation of students and the perceived IAQ, indicating a mutual interdependence between the perceptions of these two indoor environmental parameters.^39^

Thermal neutral sensation varies per individual^33^ and depends also on many factors, for example, climate, cooling or heating season, adaptation period, and room temperature at home. When the thermal environment is assessed, there is evidence that indicates gender differences. Female students tend to feel cold more than male students.^24^, ^25^, ^33^, ^36^, ^41^ High indoor temperatures increase students' heart rate.^27^, ^31^ Liu et al^46^ also observed this effect. They concluded that these high temperatures strongly stimulate the sympathetic nervous system, causing thermal discomfort. Furthermore, a heart rate that exceeds the normal heart rate at rest may affect students' cognitive performance negatively.^27^

The included studies indicate that a thermal neutral sensation will occur at different indoor temperatures, between 19.5 and 23.3°C,^27^, ^36^, ^39^ and depends on outdoor temperature and transition period.^24^, ^25^, ^39^ However, De Abreu‐Harbich et al,^47^ for example, observed a thermal neutral sensation at an even higher indoor temperature of 25.9°C, among students in a high‐altitude tropical climate. Furthermore, thermal neutrality is influenced by more factors, besides indoor and outdoor temperature, transition period and climate. For example, indoor air humidity and the clothing insulation (clo) value, metabolic rate, gender, and age of students will influence their thermal sensation vote.^48^ Even students' socio‐economic and socio‐cultural background will influence their thermal sensation vote.^49^ This explains why students' thermal sensation will differ, even among students in the same classroom and in the same indoor environment. In line with the findings of Singh et al,^49^ students in university classrooms report feeling comfortable on the cooler side of the thermal sensation scale. In order to assess thermal sensation, combined thermal sensation scales (eg, a combined scale for the thermal sensations “slightly warm” and “warm”) should be avoided because all descriptors of human thermal sensation can cause a different effect on perceived or measured short‐term academic performance.^28^

Perceived visual comfort can be positively influenced with different correlated color temperatures. Warm white light provides a relaxing environment and supports communication, and should gradually change to blue‐enriched white light after its prolonged use during the morning to prevent drowsiness and dozing off in class.^32^ Application of these different correlated color temperatures imitates the natural change of daylight during the day and therefore supports teachers' and students' circadian rhythm.^30^ Application of a lighting system with a color temperature of 4000K in classrooms can also influence the ability to concentrate positively.^29^ Although artificial lighting systems are necessary for creating optimal lighting conditions for facilitating in‐class activities, students should be always provided with access to daylight in order to regulate students' circadian rhythm and level of stress hormones, that is, cortisol.^30^ And according to Reid and Zee,^50^ regulation of students' circadian rhythm is important because it influences students' alert state and cognitive performance.

Acoustic comfort is an important factor, which might play a dominant role in how the overall IEQ is perceived by students.^41^ Creating acceptable acoustic conditions in classrooms is important. Poor acoustic conditions can affect students' ability to hear the teacher.^42^ Furthermore, Persinger et al^51^ pointed out that poor acoustic conditions can cause mental health effects such as fatigue and concentration problems among students. In addition to what we have elaborated before, it is essential for students to hear the voice of teachers clearly in order to be able to learn effectively.

The evidence presented suggests that the IEQ influences the quality of learning. By providing conditions in which students feel comfortable, they will be able to concentrate better and keep their attention for a longer period of time. However, poor IEQ can cause negative health effects, such as fatigue and sleepiness in students. These effects can lead to sick leave, which in turn can affect students' achievement.^15^

### 4.3 The influence of the IEQ on students' academic achievement

The focus of all included studies, which examined the effect of IEQ on students' academic achievement, was on students' short‐term academic performance. Therefore, the impact of the IEQ on students' long‐term academic performance could not be determined yet. Further research is needed to determine the possible relation between the short‐term and long‐term academic performance of students.

Nine of the included IAQ‐studies^22^, ^23^, ^26^, ^29^, ^30^, ^31^, ^35^, ^39^, ^41^ used CO_2_ concentration in ambient air as the performance indicator of the IAQ. One may assume, however, as humans (generally) are the single source of CO_2_, both CO_2_ concentration and other bio‐effluents are correlated. None of the included studies analyzed the effect of pure elevated CO_2_. Therefore, the observed effect of CO_2_ on improved or impaired short‐term academic performance is caused by a combination of CO_2_ and other pollutants. The reported CO_2_ concentration in the identified studies should be considered as an indicator of ventilation adequacy, which can be related to human bio‐effluents, but also to material emissions, chemicals used indoors, as well as other indoor sources of pollutants. The negative effect of elevated concentrations of bio‐effluents, but not pure CO_2_, and other constituents on cognitive performance is also confirmed by Zhang et al^52^ The cognitive performance of students can decline by as much as 13% (*P* < 0.001) when the CO_2_ concentration increases from 600 to 1000 ppm.^22^, ^23^ However, this concentration of CO_2_ still meets prevailing guidelines.^53^ High CO_2_ concentrations of 1800 ppm might affect cognitive performance with 24% (*P* < 0.001).^22^, ^23^ The influence of high CO_2_ concentrations, as proxy for the IAQ, was higher on complex and memory‐oriented than for vigilance tasks. However, the study of Ahmed et al^22^, ^23^ is the only study that quantified the effect of IAQ on cognitive performance and examined only female students. Because of these limitations, these results need to be validated with additional field research to confirm the impact and should explore possible gender differences.

Thermal discomfort, caused by high or low temperatures, affects students' cognitive performance.^22^, ^23^, ^31^, ^33^, ^34^ Barbic et al^31^ observed a decrease of as much as 24% when students experienced thermal discomfort due to high temperature. However, not all thermal discomfort sensations lead to a deterioration of cognitive performance,^27^ and the effect is most likely task dependent. Thermal sensations “cool” and “slightly cool” can positively influence cognitive performance^28^; thermal sensations “cold,” “slightly warm,” “warm,” and “hot” can affect cognitive performance negatively.^27^ The thermal sensation “hot” affects cognitive performance of vigilance tasks and memory and learning tasks more than the thermal sensation “cold.”^22^, ^23^ Nevertheless, Bajc et al^35^ concluded that students' short‐term academic performance is not just a function of PMV index; there is no simple relation in real conditions that can link this performance to the PMV index alone.

The color temperature and light intensity of artificial lighting can affect the cognitive performance of students.^28^ This effect can be as much as 23% but this percentage is based on an average recognition rate of objects.^29^ The effect of these conditions on other cognitive tasks of students is not revealed yet.

Two studies^28^, ^42^ investigated the impact of acoustic conditions on short‐term academic performance. Xiong et al^28^ observed that under normal conditions of 22°C and an illumination level of 300‐2200 lux, an increase of sound pressure, from 40 to 70 dBA, affected overall cognitive performance negatively with 3%‐42% (*P* < 0.05). Hongisto^54^ also confirmed this effect in an office setting. As observed for thermal conditions, the extent of this effect was also task‐dependent. To quantify students' short‐term academic performance, seven of the 11 studies used standard cognitive performance tests.^22^, ^23^, ^26^, ^27^, ^28^, ^31^, ^33^, ^34^ Castro‐Martínez et al^42^ used a different method to quantify students' short‐term academic performance. They used students' examination scores on mathematics and statistics and analyzed the attention level with video recording of students' behavior in the classroom. Although an increase of reverberation time does not always lead to a decrease in short‐term academic performance, it can affect the intelligibility of background speech and therefore could influence the disturbance and performance of students.^42^, ^55^ Students' short‐term academic performance is also affected by unwanted noises in the classroom and may decrease this performance with as much as 34%.^37^

There might be a relation between perceived acoustic comfort and actual thermal conditions but the precise effect remains unclear. Research of Xiong et al^28^ revealed some relations between thermal comfort and acoustic comfort but not all cognitive tasks were affected due to a combination of these conditions. The combined effect of air temperature and CO_2_ seems to increase when air temperature and CO_2_ concentration increases according to Ahmed et al^22^, ^23^ Other factors, besides temperature, such as stress, sleep deprivation and pre‐existing disease or illness, among others, may play a role in health‐related symptoms, such as headache and tiredness.^36^ Individuals who are fatigued are also more likely to experience increased levels of psychological distress, acute health symptoms, and behavioral problems; these problems affect human performance.^22^, ^23^

It is well documented that the four individual IEQ parameters do affect the short‐term performance of students. Combined effects of thermal conditions and IAQ were observed by Ahmed^22^, ^23^ among 499 female students. In addition, they controlled the lighting and acoustic conditions. However, they did not analyze the combined influence of these conditions. Xiong^28^ analyzed the combined influence of three IEQ parameters, thermal and lighting conditions and IAQ. None of the studies analyzed the combined influence of all four indoor targeted environmental parameters. Therefore, the magnitude of the combined influence of these four IEQ parameters cannot be quantified yet.

## 5 FUTURE RESEARCH

This systematic review revealed existing evidence about the influence of the IEQ on the quality of teaching, learning, and students' short‐term academic performance. However, the influence of the IEQ on the quality of teaching could be further explored. Not only the influence of the IEQ on teachers' health, also the effect of the IEQ on the quality of instructional practices, teaching effectiveness, and the motivation of teachers should be explored. Although the short‐term academic performance has been analyzed in different studies, the relation between the IEQ and the long‐term academic performance of students was not revealed. Additional research is needed to better understand this possible relation and to quantify the impact on students' academic achievement.

For analyzing the actual environmental conditions, different measuring equipment was used and one or more key performance indicators, to determine the IEQ, were applied. Additional research is needed to determine the key performance indicators of the IEQ and how these should be measured in a classroom; in order to (consistently) relate perceptions, health symptoms, and performance to the actual IEQ. Determining key performance indicators can also contribute to making future results more comparable.

Although various standardized tests are available for measuring short‐term cognitive performance, few methods were identified for measuring the effect of the IEQ on physical health effects, emotional response, and long‐term academic performance. New methods should be developed and could help to reveal the influence of the actual and experienced IEQ on teachers' and students' health, cognitive performance, emotion, and behavior.^9^

## 6 LIMITATIONS AND STRENGTH

During the review process, all studies were assessed on quality and reliability. Assessors were the authors of this review. Each assessor examined the exact same studies. The scores of all assessors were compared and discussed and resulted in minimal differences. In addition, this process led to adjustments and fine‐tuning of the assessment procedure. This procedure was developed by the assessors and included all rubrics, as no other tool was applicable to this specific domain. Therefore, this tool can only be applied when studies related to the IEQ need to be assessed for relevance and quality. Cultural or geographical differences between the studies were not analyzed. Therefore, the optimal conditions, as presented in the collected evidence, may not be applicable in every situation and are bound to the specific cultural and geographical cultural backgrounds. However, these conditions can be used as an indication for the development of optimal indoor environment conditions for teachers and students in a specific setting.

## 7 CONCLUSION

The primary goal of this systematic literature review was to provide an overview of how classroom indoor environmental conditions influence the quality of teaching and learning and students' academic achievement in higher education. Although a wide range of relevant evidence of high‐quality research was identified, the amount of evidence which examined the effect of the IEQ on the quality of teaching is limited to only two studies on acoustics. These studies illuminate how high background noise levels affect students' ability to hear teachers' voice and increase teachers' health risks. Evidently, this is insufficient to determine the precise influence of all four IEQ parameters on teaching quality.

In this context, the first hypothesis—that the IEQ influences the quality of teaching—cannot be confirmed or rejected due to a lack of evidence. However, there is some evidence which suggests the negative impact of impaired acoustic conditions on teachers' health. The second hypothesis—that the IEQ influences the quality of learning—is confirmed. Sufficient evidence confirms that a poor IAQ, thermal, acoustic, and lighting conditions negatively influence the quality of learning due to discomfort and impaired mental and physical health of students. Moreover, optimal conditions contribute positively to the quality of learning through creating an environment in which students feel more alert and pay more attention to the information presented in the lecture. Studies showing that the IEQ influences students' academic achievement partially confirm the third hypothesis. On one side, the available evidence that specifies the influence of individual or combined indoor environmental conditions on students' short‐term academic performance is sufficient to conclude that these conditions can either influence this performance positively or negatively. Optimal IEQ conditions, in which the students performed at their best, were task‐dependent, with a preference for a relatively cool, bright, and quiet environment and in ambient air with low CO_2_ concentrations. However, on the other side, the hypothesized influence of all IEQ parameters on students' long‐term academic performance cannot be confirmed due to a lack of evidence. Therefore, the overall influence of the IEQ on students' academic achievement cannot be fully determined yet.

## CONFLICT OF INTEREST

The authors declare no competing interests.

## AUTHOR CONTRIBUTIONS

**Henk W. Brink:** Conceptualization (lead); formal analysis (lead); investigation (lead); methodology (lead); visualization (lead); writing – original draft (lead); writing – review and editing (lead). **Marcel G. L. C. Loomans:** Formal analysis (supporting); supervision (supporting); writing – original draft (supporting); writing – review and editing (supporting). **Mark P. Mobach:** Formal analysis (supporting); supervision (supporting); writing – original draft (supporting); writing – review and editing (supporting). **Helianthe S. M. Kort:** Formal analysis (supporting); supervision (lead); writing – original draft (supporting); writing – review and editing (supporting).

### Peer Review

The peer review history for this article is available at https://publons.com/publon/10.1111/ina.12745.

## Data Availability

Data sharing is not applicable to this article as no new data were created or analyzed in this study.

## Appendix 1 Overview of used keywords

**Table:**

Subject	Keywords
Higher education	Academy
	College
	Higher education
	University
Classroom	Classroom
Indoor environmental quality	Indoor built environment
	Indoor climate
	Indoor environment
	Indoor environmental quality
Indoor air quality	Carbon dioxide (CO_2_)
	Humidity (humidification)
	Hygrothermal
	Indoor air (quality)
	Outdoor air supply rate
	Particulate matter
	Ventilation
	Volatile organic compound (exposures)
Thermal conditions	PMV
	PPD
	Predicted mean vote
	Predicted percentage of dissatisfied
	Temperature
	Thermal
	Cold
	Metabolic rate
Lighting conditions	Blinding
	Fluorescent
	Glare
	Illuminance (illumination)
	Light (lighting, daylight, artificial light)
	Luminance
	Visual (conditions)
Acoustic conditions	Acoustic (acoustics)
	Noise (noisiness)
	Sound
	Reverberation
Teaching	Classroom processes
	Instructional practices
	Quality of instruction
	Teacher quality
	Teachers' use of supportive practices
	Teaching effectiveness
	Willingness of teacher
Learning	Active thinking
	Alertness
	Attention
	Concentration
	Intellectual engagement
	Learning environment
	Learning outcomes
	Learning quality
	Schoolwork
	Vigilance
Students' academic achievement	Academic achievement
	Academic outcome
	Academic performance
	Cognitive performance
	Student achievement
	Student performance
	Student success

## Appendix 2 Search strings

Search string for “quality of teaching” (Scopus):

(TITLE‐ABS‐KEY("academy" OR "college" OR "higher education" OR "university")) AND (TITLE‐ABS‐KEY("classroom")) AND (TITLE‐ABS‐KEY("indoor built environment" OR "indoor climate" OR "indoor environment" OR "indoor environmental quality") OR TITLE‐ABS‐KEY("PMV" OR "PPD" OR "predicted mean vote" OR "predicted percentage of dissatisfied" OR "temperature" OR "thermal" OR "cold" OR "metabolic rate") OR TITLE‐ABS‐KEY("acoustic*" OR "nois*" OR "sound" OR "reverbera* ") OR TITLE‐ABS‐KEY("blinding" OR "fluorescent" OR "glare" OR "illumina*" OR "light*" OR "luminance" OR "visual") OR TITLE‐ABS‐KEY("carbon dioxide" OR "CO2" OR "humidi*" OR "hygrothermal" OR "indoor air" OR "outdoor air supply rate" OR "particulate matter" OR "ventilation" OR "volatile organic compound*")) AND (TITLE‐ABS‐KEY("classroom processes" OR "instructional practices" OR "quality of instruction" OR "teacher quality" OR "teachers' use of supportive practices" OR "teaching effectiveness" OR "willingness of teacher"))

Search string for “quality of learning” (Scopus):

(TITLE‐ABS‐KEY("academy" OR "college" OR "higher education" OR "university")) AND (TITLE‐ABS‐KEY("classroom")) AND (TITLE‐ABS‐KEY("indoor built environment" OR "indoor climate" OR "indoor environment" OR "indoor environmental quality") OR TITLE‐ABS‐KEY("PMV" OR "PPD" OR "predicted mean vote" OR "predicted percentage of dissatisfied" OR "temperature" OR "thermal" OR "cold" OR "metabolic rate") OR TITLE‐ABS‐KEY("acoustic*" OR "nois*" OR "sound" OR "reverbera* ") OR TITLE‐ABS‐KEY("blinding" OR "fluorescent" OR "glare" OR "illumina*" OR "light*" OR "luminance" OR "visual") OR TITLE‐ABS‐KEY("carbon dioxide" OR "CO2" OR "humidi*" OR "hygrothermal" OR "indoor air" OR "outdoor air supply rate" OR "particulate matter" OR "ventilation" OR "volatile organic compound*")) AND (TITLE‐ABS‐KEY("active thinking" OR "alertness" OR "attention" OR "concentration" OR "intellectual engagement" OR "learning quality" OR "learning environment" OR "learning outcomes" OR "schoolwork" OR "vigilance"))

Search string for “students' academic achievement (Scopus):

(TITLE‐ABS‐KEY("academy" OR "college" OR "higher education" OR "university")) AND (TITLE‐ABS‐KEY("classroom")) AND (TITLE‐ABS‐KEY("indoor built environment" OR "indoor climate" OR "indoor environment" OR "indoor environmental quality") OR TITLE‐ABS‐KEY("PMV" OR "PPD" OR "predicted mean vote" OR "predicted percentage of dissatisfied" OR "temperature" OR "thermal" OR "cold" OR "metabolic rate") OR TITLE‐ABS‐KEY("acoustic*" OR "nois*" OR "sound" OR "reverbera* ") OR TITLE‐ABS‐KEY("blinding" OR "fluorescent" OR "glare" OR "illumina*" OR "light*" OR "luminance" OR "visual") OR TITLE‐ABS‐KEY("carbon dioxide" OR "CO2" OR "humidi*" OR "hygrothermal" OR "indoor air" OR "outdoor air supply rate" OR "particulate matter" OR "ventilation" OR "volatile organic compound*")) AND (TITLE‐ABS‐KEY("academic achievement" OR "academic outcome" OR "academic performance" OR "cognitive performance" OR "student achievement" OR "student performance" OR "student success"))

## Appendix 3 Assesment procedure for relevance and quality of study

**Table:**

	Aspect (weight factor)	0 points	1 point	2 points
Relevance of study (33%)	Scope of study (17%)	Only perceived comfort or perceived impact on performance is assessed (perception)	Physical conditions AND perceived comfort and/or perceived impact on performance is assessed (perception)	Physical conditions AND impact on performance is assessed with tests (performance)
		Number of marked rubrics:	Number of marked rubrics:	Number of marked rubrics:
	Context of study (17%)	Lab study or 'in situ' research where the IEQ in classrooms is not monitored	Lab study or 'in situ' research where the IEQ in classrooms is controlled	Lab study or 'in situ' research where multiple indoor environmental conditions in classrooms are created
		Number of marked rubrics:	Number of marked rubrics:	Number of marked rubrics:
Quality of study (67%)	Reliability (17%)	congress proceeding professional magazine	scientific journal	peer‐reviewed scientific journal
		Population/sample size not available	Population/sample size available	Population/sample size available and consist of male and female students
		Age respondents/ standard deviation not available	Age respondents are available	Age respondents and standard deviation is available
		Number of marked rubrics:	Number of marked rubrics:	Number of marked rubrics:
	Method (50%)	Method is clearly described	Method is clearly described and replicable	Method is clearly described, replicable and the study uses reliable performance tests
		Accuracy (how accurate the measurements are) of measuring equipment is not described	Accuracy (how accurate the measurements are) of measuring equipment is described	Accuracy (how accurate the measurements are) of measuring equipment is described and it is clear how the measurements were carried out in the classroom
		Zero or one indoor environmental conditions were measured (AC, TC, LC, or IAQ)	Two indoor environmental conditions were measured (AC, TC, LC, or IAQ)	Three or more indoor environmental conditions were measured (AC, TC, LC, or IAQ)
		The indoor environmental parameter is measured but it is not clearly described which performance indicator is measured	The indoor environmental parameter (AC, TC, LC, or IAQ) is measured with one performance indicator	The indoor environmental parameter (AC, TC, LC, or IAQ) is measured with two or more performance indicators
		Number of marked rubrics:	Number of marked rubrics:	Number of marked rubrics:

Relevance and Quality (RQ) score = ((Score Scope of study[Number of marked rubrics × rubric points] × 3] + Score Context of study[[Number of marked rubrics × rubric points] × 3]+Score Reliability[[Number of marked rubrics × rubric points] × 1] + Score Method[[Number of marked rubrics × rubric points] × 2.25)/36) × 100%.

## Appendix 4 General overview of all included studies

**Table:** Note

Author	Age (σ)	TC	LC	AC	IAQ	Outcomes
Ahmed^22^, ^23^	15‐22	t_a_ t_r_ v_a_= RH_i_=	E_amb_=	L_Aeq_=	CO_2_	Actual thermal sensation Thermal comfort sensations Air‐conditioner set temperature at home Self‐reported ability to focus related to thermal discomfort Self‐reported ability to focus related to other symptoms than thermal discomfort Self‐reported headache Self‐reported fatigue Self‐reported dizziness Percentages of errors for cognitive tasks
Sarbu^26^	21.17 (0.79)	RH_i_ t_a_ t_g_ t_op_ v_a_			CO_2_	Actual thermal sensation Concentrated and distributive attention
Siqueira^27^	21 (2.89)	RH_i_ t_a_ t_g_ t_r_ t_wb_ v_a_ p_a_				Verbal Reasoning Numeric Reasoning Abstract Reasoning Spatial Reasoning Mechanical Reasoning Overall Score Heart rate Blood pressure
Xiong^28^	20‐24	t_a_	E_amb_	SPL		Perception Memory Problem‐solving Attention
Yan^29^	18‐21	RH_i_ t_a_	CRI CT LF E_amb_		CO_2_	Recognition rate
Gentile^30^	16‐17	RH_i_ t_a_	E_c_ E_amb_ L_c_ L_w_ CRI CCT SPD		CO_2_	Students' mood Light perception Saliva cortisol concentration
Barbic^31^	20.07 (3.1)	t_a_	=		CO_2_	Actual thermal sensation Short‐term memory and verbal ability test Reasoning test
Choi^32^	23.53 (0.87)	RH_i_ t_a_	L_og_Ch L_og_Cy L_og_Er L_og_Ph L_og_Rh E_amb_ CT	L_Aeq_		Circadian system (melatonin and cortisol) Sleepiness perception Students' mood Visual comfort
Hoque^33^	20.7 (4.2)	RH_i_ RH_o_ t_a_ t_o_ v_a_				Actual thermal sensation Exam scores
Almaqra^34^	22.02 (0.21)	Rh_i_ = t_a_		SPL=		Cognitive performance
Bajc^35^	20‐25	RH_i_ t_a_ t_o_ t_r_ v_a_			CO_2_ CO_2_o	Concentration Productivity Actual percentage of dissatisfied Prediction of academic performance Actual thermal sensation
Mishra^24^, ^25^	18‐20	RH_i_ t_g_ t_o_ v_a_ v_ao_			CO_2_	Actual thermal sensation
Norbäck^36^	20‐25	RH_i_ t_o_			AC ACR CO_2_ N_2_O NCHO PM_10_ PSV VB VM	Sinusitis problems Perceived indoor air quality Actual thermal sensation
Shelton^37^	‐			L_Aeq_		Transfer of knowledge Lexical decision task
End^38^	20.21 (2.13)			L_Aeq_		Knowledge
Liu^39^	20 (17‐22)	CLO RH_i_ t_a_ t_g_ t_o_ t_out_ t_r_ v_a_			CO_2_ PM_2.5_	Actual thermal sensation Perceived IAQ
Rabelo^40^	29 (22‐50)			F_0_ SPL Pho CyD		Vocal intensity Percentage of phonation Cycle dose
Lee^41^	21‐30	RH_i_ t_a_ t_r_ t_op_	E_hor_	L_Aeq_ SPL	CO_2_	Actual thermal sensation Calculated occupants acceptance of the indoor environment The calculated overall acceptance of the indoor environmental quality Self‐reported learning performance
Castro‐Martínez^42^	22.4 (2.4) 21.7 (2.6)			RT		Statistics Mathematics Attention

See footnote to Table for explanation of all variables and symbols used.

σ = standard deviation.

TC = Thermal performance indicators: CLO = clothing insulation value, RH_i_ = indoor relative humidity, RH_o_ = outdoor relative humidity, t_a_ = air temperature, t_db_ = dry bulb temperature, t_f_ = floor temperature, t_g_ = globe temperature, t_mr_ = mean radiant temperature, t_o_ = mean outdoor temperature, t_op_ = operative temperature, t_r_ = radiant temperature, t_w_ = temperature of walls, t_wb_ = wet‐bulb temperature, v_a_ = air velocity.

Lighting performance indicators: Cc = contrast ((object luminance/ambient luminance)/object luminance), CCT = correlated colour temperature, CRI = colour rendering index, CT = colour temperature, DF = daylight factors, DFC = daylight factor contour, E_amb_ = ambient illuminance (illuminance), E_c_ = cylindrical illuminance, E_hor_ = horizontal illuminance, E_ver_ = vertical illuminance, L_c_ = average ceiling luminance, L_f_ = luminous Flux (lm), L_og_Ch = chloropic lux, L_og_Cy = cyanopic lux, L_og_Er = erythropic lux, L_og_Me = melanopic lux, L_og_Ph = photopic lux, L_og_Rh = rhodopic lux, L_w_ = average wall luminance, SPD = spectral power distribution.

Acoustic performance indicators: BGN = background noise or ambient noise, CyD = Cicle dose as a total quantity of complete oscillatory periods performed by the vocal folds in a set time, F_0_ = fundamental frequency, Pho = the relative time spent in phonation compared with the elapsed time monitored expressed in a percentage, RT = reverberation time, SPL = sound pressure level.

Indoor air quality performance indicators: AC = allergen concentration, ACH = air exchange rate, Cl_2_ = chlorine, CO = carbon monoxide, CO_2_ = carbon dioxide, CO_2_o = carbon dioxide outside, D = dust, Fl = flow, HCHO = formaldehyde, NO_2_ = nitrogen dioxide, O_3_ = ozone, P_a_ = partical pressure of water vapour in ambiant air, PM_10_ = particles <10 µm, PM_2.5_ = particles <2.5 µm, PSV = personal supply ventilation, SD = settled dust, SO_2_ = sulphur dioxide, TSP = total respirable suspended particulate matter, TVOC = total volatile organic compounds, VB = viable bacteria, VM = viable mouts.

“=” behind performance indicator means that condition was kept constant.
